# Cardiovascular Risk Factors in Children and Adolescents With Obesity: Sex-Related Differences and Effect of Puberty

**DOI:** 10.3389/fendo.2019.00591

**Published:** 2019-08-27

**Authors:** Chiara Guzzetti, Anastasia Ibba, Letizia Casula, Sabrina Pilia, Simona Casano, Sandro Loche

**Affiliations:** SSD Endocrinologia Pediatrica e Centro Screening Neonatale, Ospedale Pediatrico Microcitemico “A. Cao”, Azienda Ospedaliera Brotzu, Cagliari, Italy

**Keywords:** pediatric patients, obesity, cardiovascular risk factors, puberty, sex

## Abstract

**Objectives:** To evaluate the effect of gender and puberty on cardiovascular risk factors (CVRF) in obese children and adolescents.

**Methods:** One thousand four hundred and nine obese patients [age 9.7 (2.2–17.9) y; 646 Male] were studied. Subjects were stratified according to Tanner pubertal staging and age into prepubertal ≤ and >6 ys (G1 and G2), pubertal stage 2–3 (G3), and pubertal stage 4–5 (G4). Waist circumference (WC), systolic and diastolic blood pressure (SP, DP), fasting plasma glucose, insulin, post Oral Glucose Tolerance Test glucose and insulin, and lipids were evaluated. Insulin resistance was evaluated by HOMA index. Patients with no CVRF were considered metabolically healthy (MHO).

**Results:** The percentage of MHO patients was 59.8% in G1 while was consistently around 30% in the other groups. WC was more frequently abnormal in G2 males. Pubertal progression was associated with a decrease in WC abnormalities. SP was more frequently abnormal in G4 males and pubertal progression was associated with higher prevalence of abnormal SP in males. Pubertal progression was associated with an increase in hypertension rate in both sexes. HOMA was more frequently abnormal in G2 and G3 females. HDL, LDL, and TG were more frequently abnormal in G2 females. Dyslipidemia rate was higher in G2 females. Pubertal progression was associated with higher prevalence of abnormal HDL in males.

**Conclusions:** Sex and pubertal status influence the frequency of abnormalities of CVRF in obese children and adolescents. CVRF are already present in prepubertal age. Identifying patients with higher risk of metabolic complications is important to design targeted and effective prevention strategies.

## Introduction

Childhood obesity is one of the major public health problems. In recent decades, the prevalence of obesity has considerably increased worldwide due to sedentary lifestyle and high calories diets (1–3). A recent report estimated that 603.7 million of adults and 107.7 million of children worldwide are obese (3). Childhood obesity is associated with high prevalence of morbidity and mortality in adulthood (4). Early complications of obesity include abnormal glucose metabolism (hyperglycemia, insulin resistance, type 2 diabetes mellitus), dyslipidemia and hypertension which are components of the metabolic syndrome (1). Treatment of obesity is difficult and discouraging, then more effort should be addressed to prevent overweight and obesity and their complications (1).

The influence of puberty and sex on obesity related cardiovascular risk factors (CVRF) has been shown in a number of studies (5–9). In adult patients, it is known that men <50 years have a higher metabolic risk than women, and women >50 years have a higher metabolic risk than men (10, 11). The effects of sex and puberty on CVRF in children and adolescents have been reported in some studies, with conflicting results (5–7, 12–14). Puberty is characterized by changes in fuel metabolism and fat mass distribution, cholesterol, leptin, and adiponectin levels, and a reduction of insulin sensitivity (15, 16). The lifestyle changes that occur during puberty also influence the metabolic health status of these subjects. These changes are different between boys and girls (17), and hormonal changes occurring during puberty are not the only responsible factors (17–19), although the mechanisms underlying this phenomenon is not clearly understood.

The aim of this study was to evaluate the effect of sex and pubertal maturation on CVRF in a large group of children and adolescents with obesity.

## Subjects and Methods

### Subjects

Data were collected from 1,409 children and adolescents with obesity referred to our Endocrine Unit between 2000 and 2016. The study was approved by our ethic committee and written informed consent was obtained from the parents or legal guardians for all patients. All patients included in the study had simple obesity, and possible organic or genetic causes were excluded.

In all subjects height, weight, pubertal status [according to Tanner and Whitehouse (20)], waist circumference (WC), systolic and diastolic blood pressure (SP and DP, respectively) were measured. Waist circumference was measured with an anthropometric tape midway between the lower rip margin and the iliac crest at the end of a gentle expiration. Blood pressure was measured in supine position using a sphygmomanometer with appropriated sized cuff. Body mass index (BMI) was calculated as weight divided by height squared. According to the Endocrine Society guidelines (1), patients were defined obese if BMI ≥95th percentile. BMI-SDS, a more valuable tool for comparing data from patients of different age and sex, was derived from the Italian reference data (21). According to this definition, the 95th percentile corresponds to 1.65 SDS. WC percentiles (WCp) were defined for all patients according to Cook et al. (22). SP and DP percentiles were defined for all patients according to Lurbe et al. (23).

Morning serum concentrations of glucose (Gly), insulin (Ins), total cholesterol (tCHO), HDL, LDL, triglycerides (TG) were evaluated in all subjects, between 08.00 and 09.00 h, after an overnight fast, in supine position. On the same day, an oral glucose tolerance test [OGTT; 1.75 g of glucose/kg body weight, maximum 75 g; blood samples for glycemia and insulin evaluation taken at 0 and 120 min (Gly OGTT and Ins OGTT)] was performed. Homeostatic model assessment [HOMA index, Glucose (mmol/L) × Insulin (μU/L)/22.5] was used to evaluate insulin resistance.

WC was considered normal if <90th percentile (24, 25). Hypertension was defined as SP or DP ≥95th percentile (1, 26, 27). Impaired fasting glucose (IFG) was defined as glycemia ≥100 mg/dl (5.6 mmol/l) and <126 mg/dl (7 mmol/l). Impaired glucose tolerance (IGT) was defined as glycemia after OGTT ≥140 mg/dl (7.8 mmol/l) and <200 mg/dl (11.1 mmol/l). Diabetes mellitus was defined as glycemia ≥126 mg/dl (7 mmol/l) or glycemia after OGTT ≥200 mg/dl (11.1 mmol/l) (1, 28). IFG, IGT or diabetes mellitus were considered as abnormalities of glucose metabolism. tCHO was considered high if ≥200 mg/dl (5.18 mmol/l). LDL was considered high if ≥130 mg/dl (3.37 mmol/l). HDL was considered low if ≤40 mg/dl (1.04 mmol/l). TG was considered high if ≥100 mg/dl (1.13 mmol/l) in subjects younger <10 ys and ≥130 mg/dl (1.47 mmol/l) in those ≥10 ys. Dyslipidemia was diagnosed in patients with at least one abnormal lipid value (1, 27). HOMA was considered normal if <2.5 in prepubertal patients and <4 in pubertal patients (29).

Children with normal SP, DP, Gly, Gly OGTT, Ins, Ins OGTT, HOMA, tCHO, HDL, LDL, and TG were considered metabolically healthy obese patients (MHO), while children with one or more abnormal factor were considered metabolically unhealthy obese patients (MUO).

Patients were analyzed as a single group as well as after subdivision into 4 groups, according to their pubertal status and age: prepubertal ≤6 years (group 1), prepubertal >6 years (group 2), pubertal status 2–3 (group 3), pubertal status 4–5 (group 4).

### Assays

Serum glucose was determined by the glucose oxidases method (Autoanalyser, Beckman Coulter, USA). Serum insulin concentrations were measured using reagents provided by Siemens (Lianberis, UK). tCHO, LDL, HDL, and TG were measured by standard routine methods.

### Statistical Analysis

Distribution of the data was evaluated using Kolmogorov–Smirnov test. Data were presented as median and interquartile range (IQR) or count and percentage. Comparisons between 2 groups were performed using the Mann-Whitney U-test or Chi-Squared test, according to the type of variables. Comparisons between multiple groups were performed using Chi-Squared test or Kruskal-Wallis test followed by Dunn's test for *post-hoc* multiple comparison, according to the type of variables. When age distribution was not similar between males and females, and when BMI-SDS distribution was not similar between males and females and between pubertal groups, age- or BMI-SDS-adjusted ANCOVA followed by Bonferroni's test for *post-hoc* multiple comparison and logistic regression were used to compare characteristics among the study groups, according to the type of variables. Bonferroni correction was applied when appropriate. *P* ≤ 0.01 was considered significant. All statistical calculations were performed using STATISTICA version 9.1 software (StatSoft Inc., Tulsa, OK, USA) and Graph Pad Prism version 6 (GraphPad Software Inc., La Jolla, CA, USA).

## Results

Clinical and biochemical characteristics of the patients are summarized in Table 1. One thousand four hundred and nine obese children and adolescents [646 Male and 763 Female, median age 9.7 ys (range 2.2–17.9)] were included in the study. 66.8% of patients had WCp >90°. Overall, the prevalence of hypertension was 16.3%, the prevalence of abnormalities of glucose metabolism (IFG or IGT) was 7.2%, and increased insulin resistance (measured with HOMA) was present in 43.8% of patients. The prevalence of dyslipidemia (abnormalities of tCHO and/or LDL, HDL, TG) was 35%. CVRF were present even in patients ≤6 ys: 85.1% had abnormal WCp, 20.6% had dyslipidemia, 19.8% had abnormal HOMA, 5.9% had hypertension, and 3.8% had IFG or IGT. The percentage of MHO patients was 59.8% in prepubertal patients ≤6 ys while was consistently around 30% in the other groups.

**Table 1:** Clinical and biochemical characteristics of the patients.

	**All patients *n* = 1409**	**M *n* = 646**	**F *n* = 763**	***p***	**Group 1 Prepubertal ≤6 ys** ***n* = 107**	**Group 2 Prepubertal > 6 ys** ***n* = 711**	**Group 3 Pubertal Stage 2–3** ***n* = 283**	**Group 4 Pubertal Stage 4–5** ***n* = 308**	p^*^	**Significant differences between groups**
**CLINICAL CHARACTERISTICS**										
Age (ys)	9.8 (7.8–12.6)	10.2 (8.1–12.4)	9.5 (7.6–12.8)	*0.19*	5.2 (4.6–5.6)	8.5 (7.4–9.7)	11.3 (10.1–12.5)	14.3 (13.2–15.8)	***<0.0001***	1 vs. 2, 1 vs. 3, 1 vs. 4, 2 vs. 3, 2 vs. 4, 3 vs. 4
Gender (Male/Female)	646/763	–	–		44/63	373/338	149/134	80/228	***<0.0001***	1 vs. 4, 2 vs. 4, 3 vs. 4
Prepubertal/pubertal	818/591	417/229	401/362	***<0.0001***	–	–	–	–		
MHO/MUO	457/952 (48%)	216/430 (50.2%)	241/522 (46.2%)	*0.04*	64/43 (59.8%)	208/503 (29.3%)	90/193 (31.8%)	95/213 (30.8%)	***<0.0001***	1 vs. 2,1 vs. 3, 1 vs. 4
BMI-SDS	2.1 (1.9–2.3)	2.1 (1.9–2.3)	2.1 (1.9–2.3)	*0.06*	2.2 (2–2.4)	2 (1.9–2.2)	2 (1.8–2.3)	2.2 (1.9–2.6)	***<0.0001***	1 vs. 2, 1 vs. 3, 2 vs. 4, 3 vs. 4
BMI-SDS ≤2	601/1409 (42.7%)	271/646 (42%)	330/763 (43.3%)	*0.38*	29/107 (27.1%)	325/711 (45.7%)	144/283 (50.9%)	103/308 (33.4%)	***<0.0001***	1 vs. 2, 1 vs. 3,1 vs. 4, 2 vs. 4,3 vs. 4
BMI-SDS >2– ≤2.5	643/1409 (45.6%)	291/646 (45%)	352/763 (46.1%)		64/107 (59.8%)	345/711 (48.5%)	119/283 (42%)	115/308 (37.3%)		
BMI-SDS > 2.5	165/1409 (11.7%)	84/646 (13%)	81/763 (10.6%)		14/107 (13.1%)	41/711 (5.8%)	20/283 (7.1%)	90/308 (29.2%)		
**WAIST CIRCUMFERENCE**										
WCp	92 (86–96)	93 (88–97)	91 (83–96)	***<0.0001***	96 (92–98.3)	93 (88–97)	91 (83–95)	88 (77–94)	***<0.0001***	1 vs. 3, 1 vs. 4, 2 vs. 3, 2 vs. 4, 3 vs. 4
Abnormal WCp	772/1155 (66.8%)	390/541 (72.1%)	382/614 (62.2%)	***0.0004***	80/94 (85.1%)	418/577 (72.4%)	137/236 (58.1%)	137/248 (55.2%)	***<0.0001***^§^	1 vs. 3, 1 vs. 4, 2 vs. 3, 2 vs. 4
**BLOOD PRESSURE**										
SP (mmHg)	105 (95–115)	110 (100–120)	105 (95–115)	***<0.0001***	95 (85–100)	100 (90–110)	110 (100–120)	120 (110–125)	***0.0001***	1 vs. 2, 1 vs. 3, 1 vs. 4, 2 vs. 3, 2 vs. 4, 3 vs. 4
Abnormal SP	190/1358 (14%)	108/625 (17.3%)	82/733 (11.2%)	***0.001***	4/101 (4%)	69/681 (10.1%)	51/275 (18.5%)	66/301 (21.9%)	*0.013*^§^	
DP (mmHg)	60 (60–70)	60 (60–70)	60 (60–70)	*0.16*	60 (50–60)	60 (55–65)	65 (60–70)	70 (60–70)	***0.0001***	1 vs. 3, 1 vs. 4, 2 vs. 3, 2 vs. 4, 3 vs. 4
Abnormal DP	58/1358 (4.3%)	18/625 (2.9%)	40/733 (5.5%)	*0.02*	3/101 (3%)	25/681 (3.7%)	9/275 (3.3%)	21/301 (7%)	*0.04*^§^	
Hypertension	222/1358 (16.3%)	122/625 (19.5%)	100/733 (13.6%)	***0.003***	6/101 (5.9%)	88/681 (12.9%)	56/275 (20.4%)	72/301 (23.9%)	***0.008***^§^	1 vs. 3, 1 vs. 4
**GUCIDIC METABOLISM**										
Gly (mg/dl)	88 (84–93)	89 (85–93)	87 (82–91)	***<0.0001***	84 (80–90.2)	88 (83–92)	89 (84–94)	88 (84–93)	***<0.0001***	1 vs. 2, 1 vs. 3, 1 vs. 4
Gly OGTT (mg/dl)	103 (93–114)	103 (94–114)	103 (92–114)	*0.84*	93 (83–107)	103 (93.5–114)	104 (94–117)	104 (93–116)	***<0.0001***	1 vs. 2, 1 vs. 3, 1 vs. 4
Ins (mU/l)	13 (7.8–19.6)	11.9 (7.1–18)	14.3 (7.4–21.1)	***<0.0001***	7.2 (3.9–11.2)	11.6 (7–17.6)	15 (10–23.8)	17 (12.5–24)	***0.0001***	1 vs. 2, 1 vs. 3, 1 vs. 4, 2 vs. 3, 2 vs. 4
Ins OGTT (mU/l)	50.9 (32–77.7)	45.8 (29–68)	54.9 (34.7–86.2)	***<0.0001***	23.1 (16.7–38.4)	44.7 (29.2–63.8)	60.5 (40.1–101)	70.8 (47–101)	***0.0001***	1 vs. 2, 1 vs. 3, 1 vs. 4, 2 vs. 3, 2 vs. 4
IFG or IGT	101/1399 (7.2%)	48/642 (7.5%)	53/757 (7%)	*0.74*	4/106 (3.8%)	45/705 (6.4%)	30/283 (10.6%)	22/305 (7.2%)	*0.67*^§^	
HOMA	2.8 (1.6–4.3)	2.6 (1.5–4)	3.1 (1.8–4.6)	***0.0001***	1.6 (0.8–2.4)	2.5 (1.5–3.9)	3.3 (2.1–5.3)	3.6 (2.7–5.1)	***0.0001***	1 vs. 2, 1 vs. 3, 1 vs. 4, 2 vs. 3, 2 vs. 4
Abnormal HOMA	613/1398 (43.8%)	269/641 (42%)	344/757 (45.4%)	*0.19*	21/106 (19.8%)	352/705 (49.9%)	110/282 (39%)	130/305 (42.6%)	*0.4*^§^	
**LIPIDS**										
tCHO (mg/dl)	166.5 (146–188)	165 (145–185)	169 (147–190)	*0.12*	160 (149–180)	169 (148–191)	166.5 (148–186)	163 (142–184)	*0.02*	
Abnormal tCHO	213/1398 (15.2%)	89/643 (13.8%)	124/755 (16.4%)	*0.18*	9/107 (8.4%)	129/706 (18.3%)	38/282 (13.5%)	37/303 (12.2%)	*0.09*^§^	
LDL (mg/dl)	102 (86–120)	102 (86–118)	103 (87–122)	*0.2*	99 (89–116)	103 (87–124)	105 (87–120)	99 (83–118)	***0.005***	none
Abnormal LDL	215/1394 (15.4%)	85/642 (13.2%)	130/752 (17.3%)	*0.04*	10/107 (9.3%)	124/705 (17.6%)	42/279 (15.1%)	39/303 (12.9%)	*0.51*^§^	
HDL (mg/dl)	50 (43–58)	50 (43–58)	50 (43–58)	*0.43*	53 (44–60)	51 (44–59)	48 (41–56)	48 (42–55)	***<0.0001***	1 vs. 4, 2 vs. 3, 2 vs. 4
Abnormal HDL	322/1398 (23%)	126/643 (19.6%)	196/755 (26%)	***0.005***	13/107 (12.1%)	159/706 (22.5%)	75/282 (26.6%)	75/303 (24.8%)	*0.51*^§^	
TG (mg/dl)	57 (42–80)	56 (41–77)	58 (42–81.5)	*0.21*	49 (37–62)	55 (40-76)	62 (47–87.5)	64 (45–89)	***<0.0001***	1 vs. 3, 1 vs. 4, 2 vs. 3, 2 vs. 4,
Abnormal TG	112/1396 (8%)	44/643 (6.8%)	68/753 (9%)	*0.13*	6/107 (5.6%)	56/705 (7.9%)	30/281 (10.7%)	20/303 (6.6%)	*0.05*^§^	
Dyslipidemia	489/1398 (35%)	197/643 (30.6%)	292/755 (38.7%)	***0.001***	22/107 (20.6%)	255/706 (36.1%)	104/282 (36.9%)	108/303 (35.6%)	*0.51*^§^	

*Data are reported as median and IQR or count and percentage*.

* *Adjusted for BMI-SDS*.

§ *p-value refers to “pubertal groups” variable considered as an independent variable in the logistic regression model. Bold values refers to statistically significant differences among groups. Italic values were used to make p-value easily recognizable*.

Sex differences in CVRF in all patients are reported in Table 1. WCp, SP, and Gly were higher in males while Ins, Ins OGTT and HOMA were higher in females. WCp and SP were more frequently abnormal in males, while DP was more frequently abnormal in females. Hypertension was more frequent in male while dyslipidemia was more frequent in females.

Pubertal status differences in CVRF in all patients are reported in Table 1. The progression of puberty was associated with higher BMI-SDS, SP, DP, Ins, Ins OGTT, HOMA, and TG and with lower WCp and HDL. The prevalence of WCp abnormalities decreased with pubertal progression. Group 1 had lower Gly and Gly OGTT than groups 2–4.

Sex differences in CVRF in patients subdivided according to their pubertal status are reported in Table 2. BMI-SDS (groups 1 and 4), WCp (groups 2 and 4), SP (groups 3 and 4), and Gly (group 4) were higher in males. Ins, Ins OGTT, and HOMA in groups 2 and 3, and LDL and TG in group 2 were higher in females. WCp (group 2) and SP (group 4) were more frequently abnormal in males while HOMA in groups 2 and 3 and LDL, HDL, and TG in group 2 were more frequently abnormal in females. Hypertension was more frequent in group 4 male while dyslipidemia was more frequently abnormal in group 2 females.

**Table 2:** Sex and pubertal status differences in CVRF among the 4 groups.

	**Group 1** **Prepubertal ≤6 ys** ***n* = 107**			**Group 2** **Prepubertal > 6 ys** ***n* = 711**			**Group 3** **Pubertal Stage 2–3** ***n* = 283**			**Group 4** **Pubertal Stage 4–5** ***n* = 308**			**M**		**F**	
	**M** ***n* = 44**	**F** ***n* = 63**	***p^a^***	**M** ***n* = 373**	**F** ***n* = 338**	***p^b^***	**M** ***n* = 149**	**F** ***n* = 134**	***p^b^***	**M** ***n* = 80**	**F** ***n* = 228**	***p^a^***	***p^c^***	**Significant differences between groups**	***p^c^***	**Significant differences between groups**
**CLINICAL CHARACTERISTICS**																
Age (ys)	5.2 (4.6–5.5)	5.2 (4.8–5.6)	*0.51*	8.9 (7.8–10.5)	8.2 (7.2–9.1)	***<0.0001***	12.1 (11–13.2)	10.3 (9.5–11.3)	***<0.0001***	14.5 (13.8–15.8)	14.1 (13.1–15.8)	*0.08*	***<0.0001***	1 vs. 2, 1 vs. 3, 1 vs. 4, 2 vs. 3, 2 vs. 4, 3 vs. 4	***<0.0001***	1 vs. 2, 1 vs. 3, 1 vs. 4, 2 vs. 3, 2 vs. 4, 3 vs. 4
MHO/MUO	25/19 (56.8%)	39/24 (61.9%)	*0.6*	125/248 (33.5%)	83/255 (24.6%)	*0.06*	50/99 (33.6%)	40/94 (29.9%)	*0.5*	16/64 (20%)	79/149 (34.6%)	*0.015*	***0.0006***	1 vs. 2, 1 vs. 3, 1 vs. 4	***<0.0001***	1 vs. 2, 1 vs. 3, 1 vs. 4, 2 vs. 4
BMI-SDS	2.3 (2.1–2.5)	2.1 (2–2.3)	***0.001***	2 (1.9–2.2)	2 (1.9–2.2)	*0.07*	2 (1.9–2.3)	2 (1.8–2.2)	*0.11*	2.4 (1.9–2.7)	2.1 (1.9–2.5)	*0.02*	***<0.0001***	1 vs. 2, 1 vs. 3, 2 vs. 4, 3 vs. 4	***<0.0001***	2 vs. 4, 3 vs. 4
BMI-SDS ≤2	6/44 (13.6%)	23/63 (36.5%)	*0.03*	167/373 (44.8%)	158/338 (46.7%)	*0.02*	74/149 (49.7%)	70/134 (52.2%)	*0.53*	24/80 (30%)	79/228 (34.6%)	***0.008***	***<0.0001***	1 vs. 2, 1 vs. 3, 1 vs. 4, 2 vs. 4, 3 vs. 4	***<0.0001***	2 vs. 4, 3 vs. 4
BMI-SDS > 2– ≤2.5	30/44 (68.2%)	34/63 (54%)		176/373 (47.2%)	169/338 (50%)		63/149 (42.3%)	56/134 (41.8%)		22/80 (27.5%)	93/228 (40.8%)					
BMI-SDS > 2.5	8/44 (18.2%)	6/63 (9.5%)		30/373 (8%)	11/338 (3.3%)		12/149 (8.1%)	8/134 (6%)		34/80 (42.5%)	56/228 (24.6%)					
**WAIST CIRCUMFERENCE**																
WCp	97 (95–99)	96 (91–97)	*0.62*	94 (90–97)	93 (88–96)	***<0.0001***	91 (83–95)	91 (82–95)	*0.86*	93 (87–97)	83 (75–93)	***<0.0001***	***<0.0001***	1 vs. 3, 2 vs. 3	***<0.0001***	1 vs. 3, 1 vs. 4, 2 vs. 4, 3 vs. 4
Abnormal WCp	31/34 (91.2%)	49/60 (81.7%)	*0.97*	239/312 (76.6%)	179/265 (67.5%)	***0.001***	75/128 (58.6%)	62/108 (57.4%)	*0.81*	45/67 (67.2%)	92/181 (50.8%)	*0.15*	***<0.0001***^§^	2 vs. 3, 2 vs. 4	***<0.0001***^§^	2 vs. 4
**BLOOD PRESSURE**																
SP (mmHg)	100 (90–104)	90 (85–100)	*0.52*	105 (95–110)	100 (90–105)	***0.0097***	110 (105–120)	105 (100–110)	*0.14*	125 (120–135)	115 (110–120)	***<0.0001***	***<0.0001***	1 vs. 2, 1 vs. 3, 1 vs. 4, 2 vs. 3, 2 vs. 4, 3 vs. 4	***<0.0001***	1 vs. 2, 1 vs. 3, 1 vs. 4, 2 vs. 3, 2 vs. 4, 3 vs. 4
Abnormal SP	1/40 (2.5%)	3/61 (4.9%)	*0.51*	42/361 (11.6)	27/320 (8.4%)	*0.5*	35/144 (24.3%)	16/131 (12.2%)	*0.29*	30/80 (37.5%)	36/221 (16.3%)	***0.001***	***<0.0001***^§^	2 vs. 3, 2 vs. 4	*0.013*^§^	
DP (mmHg)	60 (50–65)	60 (50–60)	*0.85*	60 (55–65)	60 (54–65)	*0.24*	65 (60–70)	60 (60–70)	*0.58*	70 (65–75)	70 (60–70)	*0.96*	***<0.0001***	1 vs. 3, 1 vs. 4, 2 vs. 3, 2 vs. 4, 3 vs. 4	***<0.0001***	1 vs. 3, 1 vs. 4, 2 vs. 3, 2 vs. 4, 3 vs. 4
Abnormal DP	2/40 (5%)	1/61 (1.6%)	*0.48*	14/361 (3.9%)	11/320 (3.4%)	*0.66*	2/144 (1.4%)	7/131 (5.3%)	*0.04*	0/80 (0%)	21/221 (9.5%)	*n.a*.	*0.015*^§^		*0.04*^§^	
Hypertension	3/40 (7.5%)	3/61 (4.9%)	*0.7*	53/361 (14.7%)	35/320 (10.9%)	*0.43*	36/144 (25%)	20/131 (15.3%)	*0.72*	30/80 (37.5%)	42/221 (19%)	***0.004***	***<0.0001***^§^	none	***0.008***^§^	none
**GLUCIDIC METABOLISM**																
Gly (mg/dl)	85 (82–92)	83 (79–90)	*0.17*	89 (85–93)	87 (83–91)	*0.03*	90 (85–95)	89 (84–93)	*0.38*	91.5 (86–96)	87 (52–92)	***<0.0001***	***0.001***	1 vs. 4	***0.0002***	1 vs. 4, 1 vs. 3
Gly OGTT (mg/dl)	96 (85–107)	92 (83–103)	*0.74*	103 (94–113)	94 (104–114)	*0.03*	103 (95–115)	105 (90–117)	*0.73*	109 (94–118)	103 (93–113)	*0.63*	***0.006***	1 vs. 3, 1 vs. 4	***<0.0001***	1 vs. 2, 1 vs. 3, 1 vs. 4
Ins (mU/l)	6.6 (3.6–11.7)	5 (7.5–10.4)	*0.21*	11 (6.7–16.5)	12.7 (7.5–18.9)	***0.0001***	13.2 (8.9–20.3)	17.1 (11.1–25.6)	***<0.0001***	17.7 (12.6–23.8)	16.7 (12.6–24)	*0.73*	***<0.0001***	1 vs. 2, 1 vs. 3, 1 vs. 4, 2 vs. 3, 2 vs. 4, 3 vs. 4	***<0.0001***	1 vs. 2, 1 vs. 3, 1 vs. 4, 2 vs. 3, 2 vs. 4
Ins OGTT (mU/l)	20.5 (11.9–28.6)	25.4 (20.1–40.4)	*0.08*	41.9 (28.1–61.5)	48 (32–65)	***0.0001***	55 (39–79)	72.4 (45–117)	***0.002***	62 (46.7–94)	72.6 (47.7–102.8)	*0.32*	***<0.0001***	1 vs. 2, 1 vs. 3, 1 vs. 4, 2 vs. 3, 2 vs. 4	***<0.0001***	1 vs. 2, 1 vs. 3, 1 vs. 4, 2 vs. 3, 2 vs. 4
IFG or IGT	0/43 (0%)	4/63 (6.3%)	*0.014*	24/370 (6.5%)	21/335 (6.3%)	*0.18*	17/149 (11.4%)	13/134 (9.7%)	*0.7*	7/80 (8.8%)	15/225 (6.7%)	*0.71*	*0.07*^§^		*0.67*^§^	
HOMA	1.5 (0.7–2.6)	1.6 (0.9–2.2)	*0.24*	2.4 (1.4–3.7)	2.7 (1.5–4)	***0.0002***	2.9 (2–4.4)	3.8 (2.3–5.9)	***<0.0001***	3.8 (2.8–5.2)	3.5 (2.7–5)	*0.82*	***<0.0001***	1 vs. 2, 1 vs. 3, 1 vs. 4, 2 vs. 3, 2 vs. 4, 3 vs. 4	***<0.0001***	1 vs. 2, 1 vs. 3, 1 vs. 4, 2 vs. 3, 2 vs. 4
Abnormal HOMA	12/43 (27.9%)	9/63 (14.3%)	*0.17*	170/369 (46.1%)	182/336 (54.2%)	***<0.0001***	48/149 (32.2%)	62/133 (46.6%)	***<0.0001***	39/80 (48.8%)	91/225 (40.4%)	*0.43*	*0.64*^§^		*0.04*^§^	
**LIPIDS**																
tCHO (mg/dl)	159 (139–178)	161 (153–180)	*0.09*	166 (148–188)	173 (149–196)	*0.016*	166 (149–183)	168 (147–190)	*0.59*	162 (139–181)	163 (144–185)	*0.6*	*0.06*		***0.005***	2 vs. 4
Abnormal tCHO	3/44 (6.8%)	6/63 (9.5%)	*0.07*	58/370 (15.7%)	71/336 (21.1%)	*0.011*	19/149 (12.8%)	19/133 (14.3%)	*0.48*	9/80 (11.3%)	28/223 (12.6%)	*0.62*	*0.62*^§^		*0.09*^§^	
LDL (mg/dl)	96 (81–111)	101 (91–118)	*0.12*	102 (86–119)	106 (88–125)	***0.008***	106 (88–117)	105 (87–123)	*0.71*	99 (85–119)	100 (83–118)	*0.83*	*0.12*		***0.004***	2 vs. 4
Abnormal LDL	2/44 (4.5%)	8/63 (12.7%)	*0.19*	54/370 (14.6%)	70/335 (20.9%)	***0.003***	18/148 (12.2%)	24/131 (18.3%)	*0.65*	11/80 (13.8%)	28/223 (12.6%)	*0.96*	*0.72*^§^		*0.07*^§^	
HDL (mg/dl)	55 (44–61)	53 (44–59)	*0.54*	52 (45–59)	51 (44–59)	*0.08*	49 (41–56)	48 (41–56)	*0.31*	45 (38–52)	49 (43–56)	*0.04*	***<0.0001***	1 vs. 4, 2 vs. 4	*0.03*	
Abnormal HDL	4/44 (9.1%)	9/63 (14.3%)	*0.37*	62/370 (16.8%)	97/336 (28.9%)	***0.0001***	35/149 (23.5%)	40/133 (30.1%)	*0.4*	25/80 (31.3%)	50/223 (22.4%)	*0.17*	***0.005***^§^		*0.51*^§^	
TG (mg/dl)	49 (37–56)	48 (38–64)	*0.54*	54 (39–72)	56 (42–79)	***0.001***	59 (45–87)	63 (50–88)	*0.31*	67 (57–98)	62 (43–84)	*0.012*	***<0.0001***	1 vs. 3, 1 vs. 4, 2 vs. 4	***0.0002***	1 vs. 3, 2 vs. 3
Abnormal TG	2/44 (4.5%)	4/63 (6.3%)	*0.77*	20/370 (5.4%)	36/335 (10.7%)	***0.007***	12/149 (8.1%)	18/132 (13.6%)	*0.53*	10/80 (12.5%)	10/223 (4.5%)	*0.03*	*0.1*^§^		*0.05*^§^	
Dyslipidemia	7/44 (15.9%)	15/63 (23.8%)	*0.36*	111/370 (30%)	144/336 (42.9%)	***0.0001***	49/149 (32.9%)	55/133 (51.4%)	*0.56*	30/80 (37.5%)	78/223 (35%)	*0.9*	*0.08*^§^		*0.51*^§^	

*Data are reported as median and IQR or count and percentage*.

a *p-value refers to differences between male and female. Models are adjusted for BMI-SDS*.

b *p-value refers to differences between male and female. Models are adjusted for Age*.

c *p-value refers to differences among the 4 pubertal groups. Models are adjusted for BMI-SDS*.

§ *p-value refers to “pubertal groups” variable considered as an independent variable in the logistic regression model. Bold values refers to statistically significant differences among groups. Italic values were used to make p-value easily recognizable*.

Pubertal status differences in CVRF in patients subdivided according to sex are reported in Table 2. In both sexes, SP, DP, Ins, Ins OGTT, and HOMA increased with the progression of puberty. In males, BMI-SDS was higher in group 1, SP was lower in group 3, Gly, Gly OGTT and TG were lower in group 1, and HDL was lower in group 4. In females, BMI-SDS was higher in group 1, WCp decreased with the progression of puberty, Gly and Gly OGTT were lower in group 1, tCHO and LDL were higher in group 2 and TG was higher in group 3.

The percentage of MHO patients was higher in group 1, and WCp was more frequently abnormal in group 2, regardless of sex. Among males, SP was less frequently abnormal in group 2.

In summary:

WC was more frequently abnormal in group 2 males. Pubertal progression was associated with a decrease in WC abnormalities.
SP was more frequently abnormal in group 4 males and pubertal progression was associated with higher prevalence of abnormal SP in males. Pubertal progression was associated with an increase in hypertension rate in both males and female.
The prevalence of Gly abnormalities was similar between males and females, at any pubertal stage considered. Conversely, HOMA was more frequently abnormal in groups 2 and 3 females compared to males.
HDL, LDL, and TG were more frequently abnormal in group 2 females. Dyslipidemia rate was higher in group 2 females. Pubertal progression was associated with higher prevalence of abnormal HDL in males.
Pubertal progression was not associated with change in MHO rate, but MHO rate was higher in group 1.

## Discussion

The prevalence of childhood obesity and its complications are increasing worldwide, and, therefore, it is mandatory to recognize high risk categories on which to focus prevention interventions (1–3). Obesity related CVRF in the pediatric population are influenced by sex and pubertal status (5–9). In our group of patients with obesity, impaired insulin resistance and dyslipidemia were the most frequently diagnosed CVRF, with a prevalence of 43.8 and 35%, respectively. 16.3% had hypertension and 7.2% had abnormalities of glucose metabolism. Of note is the fact that these CVRF are already present in preschool children (≤6 years). These observation are in agreement with those of previous studies showing that dyslipidemia is frequent in children/adolescents with obesity while hypertension is rare (30–33). Conversely, Martino et al. (34) and Dalla Valle et al. (35) reported a higher prevalence of hypertension (up to 20.8 and 50% respectively).

In our study, WCp, SP, and Gly were higher in boys while Ins, Ins OGTT and HOMA were higher in girls. In order to rule out a possible effect of puberty, we evaluated the effect of sex after subdividing the group according to pubertal status. We confirmed that WCp and SP were higher in group 2 and 4 boys, while Ins, Ins OGTT and HOMA were higher in group 2 and 3 girls. Moreover, group 2 girls had higher LDL and TG levels. No sex differences were found in prepubertal patients ≤6 years. We are aware that some result of this analysis, although statistically significant, may not always reflect a biological difference. For example, 5 mmHg difference in SP between boys and girls is not clinically relevant. However, when we looked for sex differences in the prevalence of CVRF abnormalities, we confirmed a higher prevalence of abnormal WC in group 2 boys, SP and hypertension in group 4 boys and HOMA, LDL, HDL, TG and dyslipidimia in group 2. A number of studies have shown, both in the general population and in subjects with obesity, an adverse cardiometabolic profile in boys (5, 6, 14, 30, 31, 35–37), with higher WC, SP, Gly, and TG, and lower HDL (31, 36, 38), but higher Ins and HOMA in girls (35, 38). Conversely, other authors (13, 32, 34) found no sex related differences in CVRF. The mechanism underlying the sex differences in CVRF is not clearly and completely understood. Male and female have different fat distribution and metabolism (different levels of adipose tissue-derived hormones and utilization of fat stores) that may be due not only to the different gonadal hormone milieu, but also to the effect of X or Y chromosome themselves (18, 39, 40). X chromosome dosage plays a role in energy homeostasis and behavior and regulation of food intake (19, 41). Testosterone exposure during fetal life may also cause modifications of hypothalamic structure and function fundamental for energy homeostasis (39). Moreover, gender-related factors like the amount of physical activity, may contribute to differences between males and females (10). In line with these observations, we observed that sex-related differences in the prevalence of some CVRF are already present in prepubertal children with obesity (42, 43).

The physiological changes of puberty driven by the rising secretion of sex hormones are accompanied by changes in body composition, fuel metabolism, lipids levels, blood pressure and reduced insulin sensitivity (5, 9, 12, 15–17, 44). Insulin sensitivity decreases at pubertal onset, reaches lowest levels around pubertal stage 3 and then returns to prepubertal levels when pubertal maturation is completed. The transient decline in insulin sensitivity is normally accompanied by an increase of insulin secretion (7, 12, 45). This compensatory mechanism could be compromised in patients with obesity, with worsening of metabolic health status during puberty and no recovery when pubertal maturation is completed (46). Furthermore, these puberty-related modifications occur differently in boys and girls and in different ethnicities (12, 17). In our study, pubertal progression was associated with an increased prevalence of hypertension in both boys and girls, and HDL abnormalities in boys. Unexpectedly, with the progression of puberty we observed a reduction in the number of patients with abnormal WC and a near stable rate of MHO patients, regardless of sex. IFG and IGT rate and HOMA abnormalities were stable during pubertal progression.

The cross-sectional study design is a clear limitation of the study. However, the large sample size allowed to show significant differences in the prevalence and severity of CVRF related to age, sex and pubertal status. Only few studies have evaluated specifically the effect of sex and puberty on CVRF in the pediatric population excluding possible confounding factors (5–9, 12, 16, 35, 36). Furthermore, since the study was performed in a single center on a large homogeneous population of children and adolescents with obesity, all from Sardinia (Italy), the results are not influenced by ethnicity (47) or by different clinical and laboratory practices. We have not evaluated the impact of lifestyle in our cohort although we are aware that differences in lifestyle (physical activity and diet) may partially account for the sex and puberty-related differences in CVRF. Further studies are needed to clarify this point. In summary, our results show that boys tend to have higher rate of abnormal WCp and hypertension, while girls tend to have higher rate of insulin resistance and dyslipidemia. Moreover, pubertal progression was associated with a more severe obesity stage and higher hypertension rate. According to these results, we are not able to recognize a single group of patients with a higher risk of metabolic abnormalities. However, we have shown that CVRF are present in all groups, and each group have higher risk for different CVRF.

In conclusion, our study confirms that sex and pubertal status influence the prevalence and severity of CVRF in children and adolescents with obesity. CVRF are already present in prepubertal age. Identifying patients with higher risk of metabolic complications is important to design targeted and effective prevention strategies.

## Data Availability

The datasets generated for this study are available on request to the corresponding author.

## Ethics Statement

This study was carried out in accordance with the recommendations of Independent Ethical Committee of the University of Cagliari with written informed consent from all subjects. All subjects gave written informed consent in accordance with the Declaration of Helsinki. The protocol was approved by the Independent Ethical Committee of the University of Cagliari, Italy.

## Author Contributions

CG carried out the analyses, drafted the initial manuscript, and reviewed and revised the manuscript. AI, LC, SP, and SC designed the data collection instruments, collected data, and reviewed and revised the manuscript. SL conceptualized and designed the study, coordinated and supervised data collection, and critically reviewed the manuscript for important intellectual content. CG, AI, LC, SP, SC, and SL approved the final manuscript as submitted and agree to be accountable for all aspects of the work.

### Conflict of Interest Statement

The authors declare that the research was conducted in the absence of any commercial or financial relationships that could be construed as a potential conflict of interest.
